# Mechanical properties of provisional dental materials: A systematic review and meta-analysis

**DOI:** 10.1371/journal.pone.0193162

**Published:** 2018-02-28

**Authors:** Daniela Astudillo-Rubio, Andrés Delgado-Gaete, Carlos Bellot-Arcís, José María Montiel-Company, Agustín Pascual-Moscardó, José Manuel Almerich-Silla

**Affiliations:** 1 Department of Stomatology, Faculty of Medicine and Dentistry, University of Valencia, Valencia, Spain; 2 Orthodontics Teaching Unit, Department of Stomatology, Faculty of Medicine and Dentistry, University of Valencia, Valencia, Spain; 3 Preventive Dentistry Teaching Unit, Department of Stomatology, Faculty of Medicine and Dentistry, University of Valencia, Valencia, Spain; 4 Dental Pathology and Therapeutics Teaching Unit, Department of Stomatology, Faculty of Medicine and Dentistry, University of Valencia, Valencia, Spain; University of Notre Dame, UNITED STATES

## Abstract

Provisional restorations represent an important phase during the rehabilitation process, knowledge of the mechanical properties of the available materials allows us to predict their clinical performance. At present, there is no systematic review, which supports the clinicians’ criteria, in the selection of a specific material over another for a particular clinical situation. The purpose of this systematic review and meta-analysis was to assess and compare the mechanical properties of dimethacrylates and monomethacrylates used in fabricating direct provisional restorations, in terms of flexural strength, fracture toughness and hardness. This review followed the PRISMA guidelines. The searches were conducted in PubMed, Embase, Web of Science, Scopus, the New York Academy of Medicine Grey Literature Report and were complemented by hand-searching, with no limitation of time or language up to January 10, 2017. Studies that assess and compare the mechanical properties of dimethacrylate- and monomethacrylate-based provisional restoration materials were selected. A quality assessment of full-text articles were performed according to modified ARRIVE and CONSORT criteria and modified Cochrane Collaboration’s tool for in vitro studies. Initially, 256 articles were identified. After removing the duplicates and applying the selection criteria, 24 articles were included in the qualitative synthesis and 7 were included in the quantitative synthesis (meta-analysis). It may be concluded that dimethacrylate-based provisional restorations presented better mechanical behavior than monomethacrylate-based ones in terms of flexural strength and hardness. Fracture toughness showed no significant differences. Within the monomethacrylate group, polymethylmethacrylate showed greater flexural strength than polyethylmethacrylate.

## Introduction

Provisional or interim restorations are commonly used in dentistry during the time between tooth preparation and placement of the definitive restoration [1]. In view of the strong demand for good aesthetic results, provisional restorations have become a valuable tool for esthetic and functional diagnosis in dentistry. Dentists can gain their patients’ confidence by handling this intermediate stage of treatment successfully, achieving the necessary predictability for a successful final restoration [2].

Fabrication of an ideal provisional restoration is crucial for gum health and to protect the pulp, for prosthetically-guided tissue healing to achieve an acceptable emergence profile, for minimizing the migration of dental abutments, and for assessing the prospective form and function of the definitive prosthesis [2,3].

Provisional restoration materials can be divided into two groups according to their chemical composition: those based on monomethacrylates or acrylic resins, which include polymethylmethacrylate (PMMA) and polyethyl/butyl methacrylate (PEMA); and those based on dimethacrylates or bis-acryl/composite resins such as bisphenol A-glycidyl dimethacrylate (Bis-GMA) and urethane dimethacrylate (UDMA; these resins are polymerized by light) [3]. The technology of provisional restoration materials has evolved in recent years, giving rise to improvements in the basic chemical composition that have made it possible to obtain commercial products which can be used with direct techniques, with good clinical and mechanical performance. This improves clinical practice, saves time and money and avoids sending to the laboratory for provisional restoration manufacture by indirect techniques [3].

Provisional restorations are subjected to chewing forces and require specific mechanical properties that allow them to survive the repeated functional forces of the oral environment, so in order to predict the behavior of a material, it is important to understand its mechanical properties. In clinical settings such as changes in the vertical dimension in full oral rehabilitation, long-span fixed prostheses, temporomandibular joint dysfunction therapies or patients who exhibit parafunctional habits, the mechanical properties of intermediate restorations play an important role in enabling the dentist to assess commercial products critically and choose the ideal material for a specific clinical situation [2].

Several in vitro studies have examined the mechanical properties of provisional restoration materials used with direct techniques. The aim of the present study is to compare and assess the available evidence through a systematic review and meta-analysis of the literature, seeking to answer the following research question: *In provisional restorations executed using direct techniques*, *do the mechanical properties of dimethacrylate-based materials differ from those of monomethacrylate-based materials in terms of flexural strength*, *fracture toughness and hardness*?

## Materials and methods

This systematic review was conducted in accordance with the PRISMA (Preferred Reporting Items for Systematic Reviews and Meta-Analyses) guidelines [4]. (S1 Table).

### Search strategy

An exhaustive bibliographic search was conducted in MEDLINE-PubMed, Embase, Web of Science (Core Collection) and Scopus to identify relevant articles published up to 10 January 2017 with no limitations on the language or year of publication. Controlled vocabulary (MeSH terms in Pubmed and Emtree terms in Embase) and free-text terms in the titles and/or abstracts were used to define the search strategy in all the databases. The search strategies were implemented with keywords based on each section of the PICO question, separated by the Boolean operator OR, then all the sections were combined using the Boolean operator AND. The grey literature search was conducted in the New York Academy of Medicine Grey Literature Report. The electronic database searches were carried out by two of the authors (DAR and ADG), working separately. The keywords used were: **MesH term**s: “dental restoration, Temporary,” “tooth crown”, “denture, partial, Temporary”, “polymethyl methacrylate”, "bisphenol a-glycidyl methacrylate", “mechanical phenomena”, “mechanical processes”; **Emtree terms**: “tooth prosthesis”, “tooth crown”, “fixed partial denture”, “poly(methyl methacrylate)”, “mechanics”, “mechanical stress”, “hardness”. **Free Terms:** provisional dental restoration, interim dental restoration, temporary dental restoration, provisional crown, interim crown, temporary crown, provisional partial fixed prosthesis, PMMA, PEMA, bis-acryl, provisional resin, interim resin, mechanical properties, fracture toughness, flexural strength. (Table 1).

**Table 1:** Eletronic databases and research strategies.

**Pubmed** **P-I #1** ((((((((((((("dental restoration, temporary"[mesh terms] or "tooth crown"[mesh terms]) or "denture, partial, temporary"[mesh terms]) or (provisional[all fields] and dental restoration[title/abstract])) or (interim[all fields] and dental restoration[title/abstract])) or temporary dental restoration[title/abstract]) or provisional crown[title/abstract]) or temporary crown[title/abstract]) or interim crown[title/abstract]) or (provisional[all fields] and partial[all fields] and fixed partial[title/abstract])) or (interim[all fields] and partial[all fields] and fixed partial[title/abstract])) or (temporary[all fields] and partial[all fields] and fixed partial[title/abstract])) and hasabstract[text]) and **C #2** (((((("polymethyl methacrylate"[mesh terms] or "bisphenol a-glycidyl methacrylate"[mesh terms]) or pmma[title/abstract]) or bis-acryl[title/abstract]) or (interim[all fields] and resin[title/abstract])) or provisional resin[title/abstract] and hasabstract[text]) and hasabstract[text])) **O #3** ((((((("mechanical phenomena"[mesh major topic] or "mechanical processes"[mesh major topic]) or "dental restoration wear"[mesh major topic]) or mechanical properties[title/abstract]) or fracture toughness[title/abstract]) or flexural strength[title/abstract]) or surface wear[title/abstract]) and hasabstract[text]) and hasabstract[text] **#1 AND #2 AND #3**
**Embase** **P-I #1** ‘tooth prosthesis’/exp OR ‘tooth prosthesis’ OR ‘tooth crown’/exp OR ‘tooth crown’ OR ‘fixed partial denture’/exp OR ‘fixed partial denture’ OR ‘dental surgery’/exp OR ‘dental surgery’ OR ‘provisional dental restoration’ OR ‘interim dental restoration’ OR ‘temporary dental restoration’ OR ‘provisional crown’ OR ‘temporary crown’ OR ‘interim crown’ OR ‘provisional partial fixed partial’ OR ‘interim partial fixed partial’ OR ‘temporary partial fixed partial’ AND [embase]/lim **C #2** ‘poly(methyl methacrylate)’/exp OR ‘poly(methyl methacrylate)’ OR ‘bisphenol a bis(2 hydroxypropyl) ether dimethacrylate’/exp OR ‘bisphenol a bis(2 hydroxypropyl) ether dimethacrylate’ OR ‘pmma’ OR ‘bis acryl’ OR ‘provisional resin’ OR ‘interim resin’ AND [embase]/lim **O # 3** ‘mechanics’/exp OR ‘mechanics’ OR ‘mechanical stress’/exp OR ‘mechanical stress’ OR ‘hardness’/exp OR ‘hardness’ OR ‘mechanical properties’ OR ‘fracture toughness’ OR ‘flexural strength’ OR ‘surface wear’ AND [embase]/lim **#1 AND #2 AND #3**
**Web of Science (Core Collection)** **P-I #1** TS = (Provisional dental restoration* OR Interim dental restoration OR Temporary dental restoration OR Provisional Crown OR Temporary Crown OR Interim crown OR Provisional partial fixed partial OR Interim partial fixed partial OR Temporary partial fixed partial) *Indexes = SCI-EXPANDED*, *SSCI*, *A&HCI*, *CPCI-S*, *CPCI-SSH*, *ESCI*, *CCR-EXPANDED*, *IC Timespan = All years* **C #2** TS = (poly methyl methacrylate OR PMMA OR Bisphenol A-Glycidyl Methacrylate OR bis-acryl OR (poly methyl methacrylate AND bis-acryl) OR provisional resin OR interim resin) *Indexes = SCI-EXPANDED*, *SSCI*, *A&HCI*, *CPCI-S*, *CPCI-SSH*, *ESCI*, *CCR-EXPANDED*, *IC Timespan = All years* **O #3** TS = (mechanical properties OR fracture toughness OR flexural strength OR surface Wear OR dental restoration wear OR hardness) *Indexes = SCI-EXPANDED*, *SSCI*, *A&HCI*, *CPCI-S*, *CPCI-SSH*, *ESCI*, *CCR-EXPANDED*, *IC Timespan = All years* **#1 AND #2 AND #3**
**Scopus** **P-I #1** (TITLE-ABS-KEY (provisional dental restoration) OR TITLE-ABS-KEY (interim dental restoration) OR TITLE-ABS-KEY (temporary dental restoration) OR TITLE-ABS-KEY (provisional crown) OR TITLE-ABS-KEY (temporary crown) OR TITLE-ABS-KEY (interim crown) OR TITLE-ABS-KEY (provisional partial fixed partial) OR TITLE-ABS-KEY (interim partial fixed partial) OR TITLE-ABS-KEY (temporary partial fixed partial)) **C #2** (TITLE-ABS-KEY (polymethyl methacrylate) OR TITLE-ABS-KEY (pmma) OR TITLE-ABS-KEY (bisphenol a glycidyl methacrylate) OR TITLE-ABS-KEY (bis-acryl) OR (TITLE-ABS-KEY (polymethyl methacrylate) AND TITLE-ABS-KEY (bis-acryl)) OR TITLE-**ABS-KEY (provisional resin) OR TITLE-ABS-KEY (interim resin))** **O #3** ((TITLE-ABS-KEY (mechanical properties) OR TITLE-ABS-KEY (fracture toughness) OR TITLE-ABS-KEY (flexural strength) OR TITLE-ABS-KEY (surface wear) OR TITLE-ABS-KEY (hardness))) **#1 AND #2 AND #3**
New York Academy of Medicine Grey Literature Report *Provisional dental restoration*, *Interim dental restoration*, *Temporary dental restoration*, *Provisional Crown*, *Temporary Crown*, *Interim crown*, *Provisional partial fixed partial*, *Interim partial fixed partial*, *Temporary partial fixed partial* additional keyword *Polymethyl methacrylate Pmma*,*Bisphenol a glycidyl methacrylate*, *Bis-acryl*, *Mechanical properties*, *Fracture toughness*, *Flexural strength*, *Surface Wear*, *Hardness*.

Abbreviations: PICO Strategy: P: Population, Intervention: Comparator, O: Outcome

### Eligibility criteria

Studies that analyzed and compared the mechanical properties of provisional restoration materials for use with direct techniques or in the dentist’s office, included both basic chemical composition groups (monomethacrylates and dimethacrylates) in the comparison, were included in the review.

The exclusion criteria covered articles that investigated provisional restoration materials for heat-curing or fabrication by indirect techniques, or compared the mechanical properties of different materials within a single chemical composition group, or studied provisional restorations placed on implants, provisional restorations in relation to endodontics or provisional cements. Case reports, case series, techniques for fabricating provisional restorations, literature reviews, abstracts, interviews, editorials and expert opinions were also excluded.

### Screening and selection

The studies located in the searches were screened in duplicate, independently, by two researchers (DAR and ADG) in order to identify those with titles and abstracts that met the inclusion criteria. The articles on which both authors agreed were selected.

The full text of the articles selected on their titles and abstracts were read and the modified ARRIVE and CONSORT [5] criteria were applied to assess the methodological quality of the article as regards correct conduct and the structure of title, abstract, introduction, methods, results, discussion and conclusions (S2 Table). The references listed in all the articles selected after reading the full text were reviewed manually and checked against the inclusion criteria. Disagreements concerning their inclusion were resolved through discussion with the third author (APM).

### Data extraction

A data extraction protocol was defined and assessed by two of the authors (DAR and ADG). The data were extracted independently from the full-text articles selected for inclusion, using a standardized form in electronic format (Office Excel 2011 software, Microsoft Corporation, Redmond, WA, USA). The authors classified the information on authors/year, study design, sample size, chemical composition, mechanical property studied, results, conclusions and risk of bias.

### Assessment of risk of bias

The assessment of risk of bias in the in vitro studies included in this review was based on a previous study [6], and verified whether the mechanical properties were analyzed in accordance with the following parameters: (a) standardization of sampling procedures, (b) single operator, (c) description of sample size calculation, (d) blinding of test machine operator, and (e) calibration of sample size before applying the test, test design, and flexural strength, fracture toughness and hardness calculations in accordance with standards and specifications.

If the article reported clearly on the parameter it received a score of 0 for that specific parameter, if a particular parameter was reported but insufficiently or unclearly the score was 1, and if it was not possible to find this information the score was 2.

Articles that scored between 0 and 3 were classified as being at low risk of bias, those with scores of 4 to 7 as moderate-risk, and scores of 8 to 10 as high-risk.

The risk of bias of the in vitro studies included in the review was assessed independently, in duplicate, by two authors, and any disagreement on the evaluation was resolved by consensus.

To control for publication bias, the fail-safe number was used. This indicates the number of non-significant studies that would be required for the observed significance to disappear.

### Data analysis

Given the considerable heterogeneity of the studies as regards research design, methods used, sample sizes, storage, and generation of materials, only studies that met certain criteria were included in the meta-analysis. For flexural strength, the criteria were: moderate or low risk of bias; compliance with ISO 10477:2004 or ADA 27, or specimens measuring 25 x 2 x 2 mm stored in a wet medium for between 24 hours and 14 days, and calibration of a universal testing machine with a crosshead speed of 0.75–1 mm/min; reporting the means and standard deviations for the different groups; and dimethacrylate group materials of a generation currently in use. For fracture toughness the criteria were: moderate or low risk of bias, compliance with ISO 13586:2000, or specimens geometry following the single-edge notched or compact tension methods, stored in a wet medium for between 24 hours and 14 days; reporting the means and standard deviations for the different groups; and dimethacrylate group materials of a generation currently in use. For the property of hardness, the criteria were analysis of Vickers or Knoop hardness in a wet medium, and reporting the mean values and standard deviations.

For the meta-analysis, the difference in means between the two groups in the studies included in this analysis was determined. The random effect model was considered when the heterogeneity was below 1. Heterogeneity was assessed by the Q test, for p < 0.1, as well as by the I^2^ test. For the combination of studies, a random effects method was used to calculate the differences in means. When three or more studies were included in the meta-analysis, a funnel plot and the fail-safe number were used to analyze publication bias. The meta-analysis was carried out with the random effects model of the Comprehensive Meta Analysis V3 software application.

Meta-analyses were conducted for flexural strength, fracture toughness and Knoop hardness alone, as the articles that studied Vickers hardness provided insufficient data and lacked the necessary information for a meta-analysis. Consequently, Vickers hardness was only included in the systematic review and descriptive analysis.

## Results

### Search and selection

**Fig 1:**
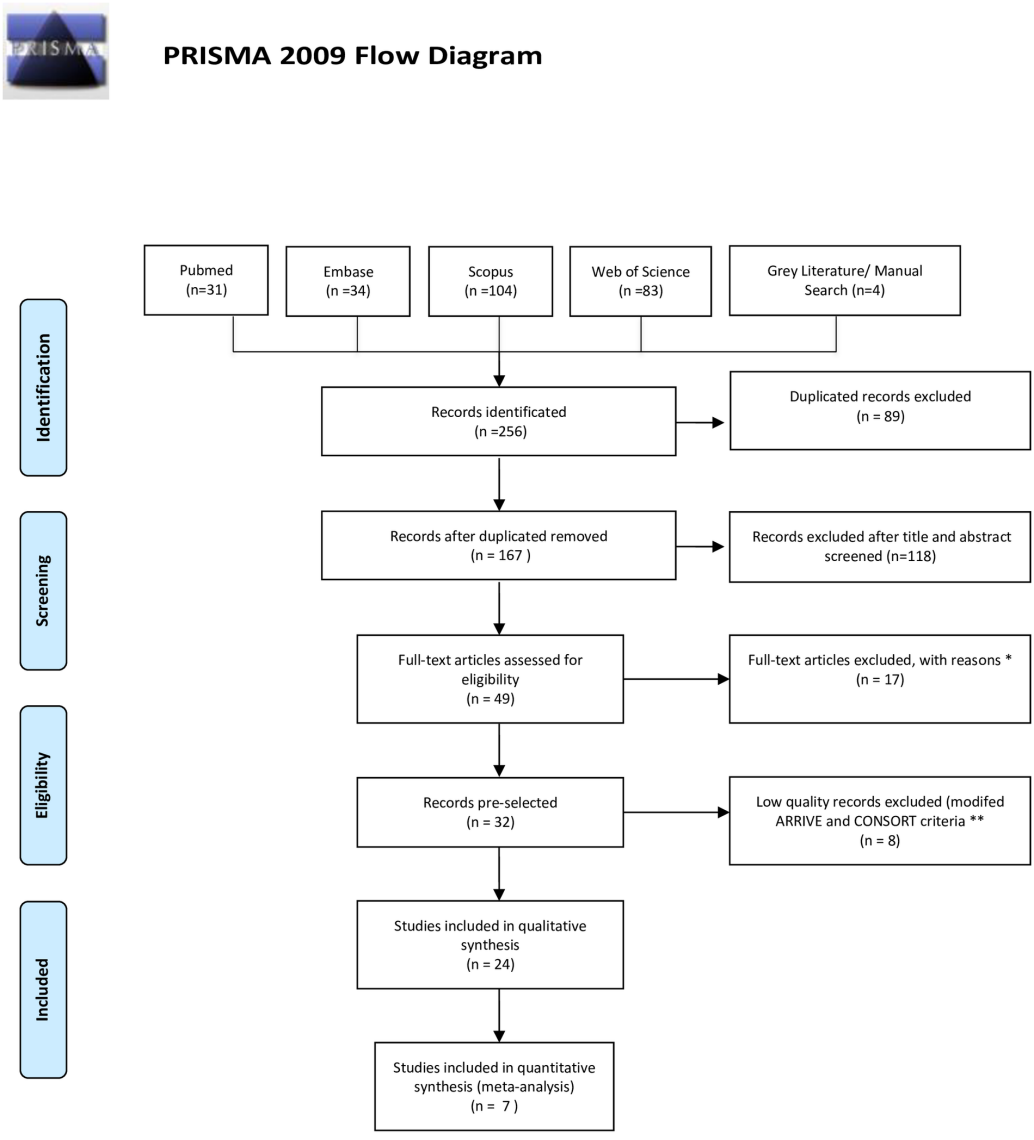
The PRISMA flow diagram. From Moher D, Liberati A, Tetzlaff J, Altman DG, The PRISMA Group (2009). Preferred Reporting Items for Systematic Reviews and Meta-Analyses: The PRISMA Statement. PLoS Med 6(7): e1000097. doi:10.1371/journal.pmed1000097. *: 5 only studied monomethacrylates, 3 only studied dimethacrylates, 3 studied provisional restoration repair, 2 used thermoplastic polyester, 1 examined provisional cement, 1 assessed reinforcing monomethacrylate materials with fiberglass, and 2 were narrative literature reviews **: Incomplete description of materials, methods or details of the experimental procedure, difficult to replicate the test, not following standardized test procedures, not describing bias reduction strategies, no calculation of sample size, incomplete statistical analysis, no conflict of interests statement, Limited interpretation and comparison of results with the available literature. **For more information, visit** www.prisma-statement.org.

The PRISMA statement flowchart summarizing the selection process is shown in Fig 1. The search returned 256 studies. Of these, 89 duplicates were excluded. Another 118 studies were excluded because they did not meet the eligibility criteria. The remaining 49 studies were selected for examination of the full text, which resulted in excluding 17 articles which did not meet the inclusion criteria. Subsequently, 8 of the remaining 32 articles were excluded after modified ARRIVE and CONSORT criteria were applied. Of the remaining 24 studies included in the systematic review, 15 studied flexural strength, 3 studied fracture toughness, 2 studied hardness, 1 studied flexural strength and fracture toughness and 2 studied flexural strength and hardness. Only 1 studied all three properties together. (Table 2).

**Table 2:** Summary of the studies included in the systematic review.

Author, year	Type of study	n	Property	Chemical composition	Results [SD]	Conclusions
Abdulmohsen et al[10] (2016)	In vitro	12 per material	FS	Bis-acryl PEMA	113.6 (8.5) MPa 33.7 (2.5) MPa	Dimethacrylate greater flexural strength, monomethacrylate (PEMA) greater exothermic reaction
Rayyan et al[24] (2015)	In vitro	5 per material per test	FS	Bis-acryl PMMA PMMA CAD/CAM	118 (8) MPa 111 (9) MPa 142 (12) MPa	CAD/CAM tooled provisional crowns showed better color stability and physical and mechanical properties than those made with conventional techniques
Penate et al[25] (2015)	In vitro	10 per material per reinforcement	FS	Bis-acryl PMMA PEMA PMMA CAD/CAM	208.9 (61.6)N 340.7 (75.6) N 227.4 (105) N 515.8 (21.3) N	Flexural strength greater in monomethacrylates, fiberglass reinforcement best mechanical properties
Thompson and Luo[13] (2014)	In vitro	10 per material	FS FT H	Bis-acryl PMMA Bis-acryl PMMA Bis-acryl PMMA	88.73 MPa 47.62 MPa 1.90 KJ/m^2^ 1.41 KJ/m^2^ 9.44 VHN 16.67 VHN	Flexural strength and fracture toughness greater in bis-acryl, surface hardness greater in PMMA.
Yanikoğlu et al[23] (2014)	In vitro	5 per material per simulation solution	FS	Bis-acryl PMMA	115.91 MPa 71.2 MPa	Provisional materials. Bis-acryl showed greater flexural strength than methacrylate resins.
Hamza et al [30] (2014)	In vitro	N/S	FS	Bis-acryl PMMA PEMA	61.6 (8.4) MPa 52.9 (7.6)MPa 16.3 (3.5) MPa	Provisional materials. Bis-acryl showed greater flexural strength than methacrylate resins. Interaction with POSS depended on resin chemistry.
Poonacha et al[14] (2013)	In vitro	35 per material	FS	Bis-acryl (self) Bis-acryl (photo) PMMA	27.20 (1.7)MPa 37.20 (3.2)MPa 56.2 (6.4)MPa	Monomethacrylate presented flexural strength
Jo et al[9] (2011)	In vitro	10 per material	FS H	Bis-acryl (self) Bis-acryl (photo) PMMA (self) Bis-acryl (self) Bisa-cryl (photo) PMMA (self)	77.97 (1.19) MPa 58.81 (1.19) MPa 51.89 (0.83) MPa 12.6 (0.33) KHN 15.9 (0.26) KHN 10.01 (0.34) KHN	Dimethacrylates presented greater flexural strength and hardness than monomethacrylates
Alt et al[20] (2011)	In vitro	10 per material	FS	Bis-acryl CAD/CAM Bis-acryl direct PMMA CAD/CAM PEMA CAD/CAM PEMA direct	875.8 (145) N 268.4 (101) N 325.2 (86.4) N 264.0 (38.2) N 138.5 (54.4) N	CAD/CAM temporary restorations present high flexural strength
Zortuk et al[15] (2010)	In vitro	10 per material	FS	Bis-acryl PMMA	353.8 (14.79)N 581.9 (136.73) N	Monomethacrylate presents greater fracture resistance
Nejatidanesh et al[7] (2009)	In vitro	10 per material	FS	Bis-acryl (self) Bis-acryl (dual) PMMA (self) PMMA (photo) PEMA	70.50 (6.74) MPa 94.69 (12.06) MPa 55.13 (6.91) MPa 40.90 (5.16)MPa 40.59 (6.18) MPa	Dimethacrylate presented higher flexural strength values
Balkenhol et al[21] (2009)	In vitro	10 per material per storage environment	FT	Bis-acryl PEMA	1 MPa.m^1/2^ 0.7 MPa.m^1/2^	Monomethacrylates presented greater fracture toughness during the first 30 min owing to plastic deformation before fracture. After that time, dimethacrylates possessed greater fracture toughness
Balkenhol et al[18] (2008)	In vitro	10 per material	FS	Bis-acryl (self) Bis-acryl (dual) PEMA	67.5 (8.1) MPa 122.8 (6.4) MPa 35.8 (0.9) MPa	Dual-cure dimethacrylates presented greater flexural strength up to 72 h
Kim and Watts[11] (2007)	In vitro	7 per material	FS	Bis-acryl PEMA	1010 N 548.62 N	Dimethacrylates presented greater fracture resistance at edge of specimen
Akova et al[22] (2006)	In vitro	10 per material	FS H	Bis-acryl PEMA Bis-acryl PEMA	101.4 (9.45) MPa 74.1 (7.9) MPa 9.6 (1.65) KHN 7 (0.9) KHN	Flexural strength and hardness are influenced by simulation solutions
Kim and Watts[27] (2004)	In vitro	5 per group	FT	Bis-acryl PEMA	2.5 (0.13)MPa.m^1/2^ 1.4 (0.1) MPa.m^1/2^	Dimethacrylate presented greater fracture toughness than monomethacrylate. Glass fiber reinforcement increased the fracture toughness of both materials. Storage affected fracture toughness
Hamza et al[28] (2004)	In vitro	5 per group	FS FT	Bis-acryl PMMA PEMA Bis-acryl PMMA PEMA	62.33 (8.51) MPa 52.88 (4.96) MPa 16.34 (3.48) MPa 0.87(0.05) MPa.m^1/2^ 1.25(0.06) MPa.m^1/2^ 0.62(0.07) MPa.m^1/2^	Reinforcement and surface treatment of fibers is an effective method for increasing fracture toughness and flexural strength.
Yap et al[12] (2004)	In vitro	6 per material per storage environment	H	Bis-acryl (self) Bis-acryl (photo) Bis-acryl (dual) PMMA (self) PMMA (photo)	12.43 (0.28) KHN 8.78 (0.63) KHN 13.43 (0.56) KHN 10.18 (0.67) KHN 11.32 (1.06) KHN	Dimethacrylate more resistant to damage by dietary simulating solvents
Lang et al[16] (2003)	In vitro	10 per material per storage time	FS	Bis-acryl PMMA	829 N 525.5 N	Dimethacrylate presented higher fracture resistance values
Haselton et al[8] (2002)	In vitro	10 per material	FS	Bis-acryl PMMA PEMA	102.7 (14.4) MPa 83.1 (5.3) MPa 89.9 (20.1) MPa	No correlation between flexural strength and type of provisional dental resin
Ireland et al[19] (1998)	In vitro	13 per material per storage time	FS	Bis-acryl (dual) Bis-acryl (Photo) PEMA	72.39 MPa 75.33 MPa 52.88 MPa	Dual-cured dimethacrylate presented greatest flexural strength at 24 h.
Samadzade et al[29] (1997)	In vitro	10 per material per reinforcement	FS	Bis-acryl PMMA	46.59 Kg. 49.86 Kg.	Polyethylene fiber reinforcement increases flexural strength
Gegauff and Wilkerson[26] (1995)	In vitro	7 per material per storage environment	FT	Bis-acryl (photo) PMMA PEMA	0.79 MPa.m^1/2^ 1.22 MPa.m^1/2^ 0.7 MPa.m^1/2^	Photo-cured dimethacrylate presented greater fracture toughness than monomethacrylate.
Diaz-Arnold et al[17] (1990)	In vitro	5 per material	H	Bis-acryl PMMA	17.43 (1.63) KHN 14.0 (0.6) KHN	Dimethacrylates have greater surface hardness owing to their chemical composition

Abbreviations: SD: standard deviation; N/S: not stated; FS: flexural strength; FT: fracture toughness; H: hardness,; PMMA: polymethylmethacrylate; PEMA: polyethylmethacrylate; self: self-cured; photo: light-cured; dual: chemical/light-cured; MPa: megaPascal; MPa.m^1/2^ or KJ/m^2^: critical stress intensity factor; N: Newton; VHN: Vickers Hardness Number; KHN: Knoop Hardness Number; min: minutes; h: hours

### Risk of bias

**Fig 2:**
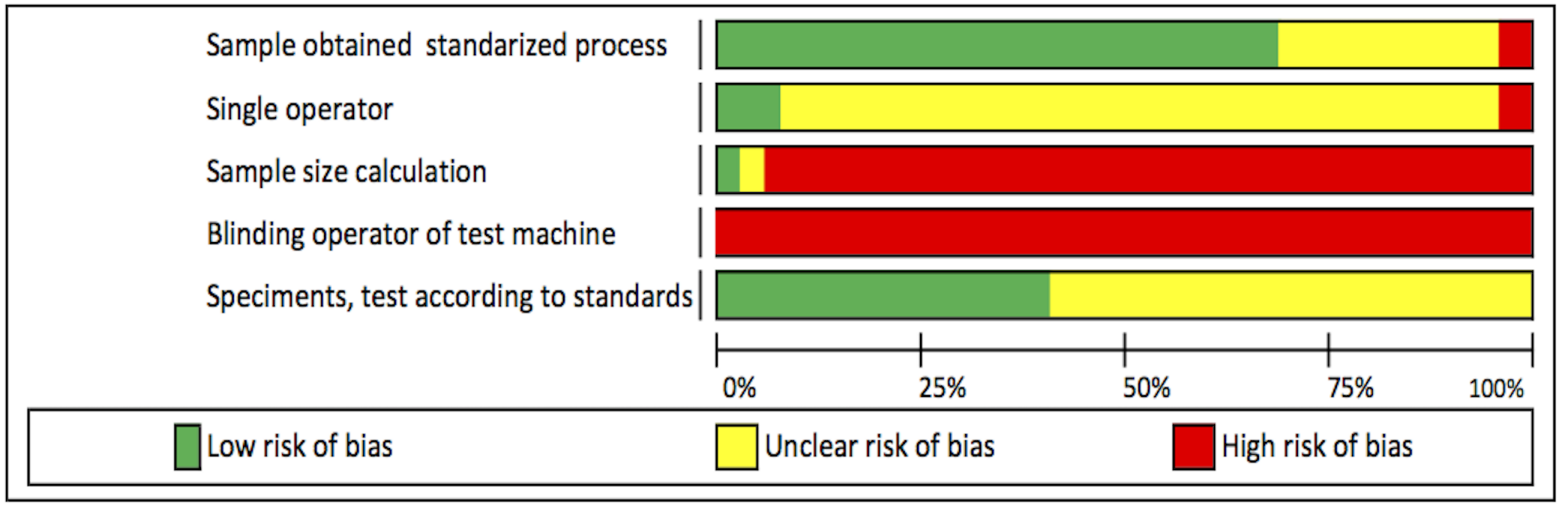
Summary of the risk of bias assessment. From Aurelio IL, Marchionatti AM, Montagner AF, May LG, Soares FZ. Does air particle abrasion affect the flexural strength and phase transformation of Y-TZP? A systematic review and meta-analysis. [6].

All of the 24 studies included in the systematic review presented moderate risk of bias. No high risk of bias was found in any of the articles included. (Fig 2 and Table 3).

**Table 3:** Risks of bias of the studies evaluating mechanical properties.

Author/Year	Samples obtained through a standardized process	Single operator of the machine	Sample size calculation	Blinding of the testing machine operator	Specimens, test, and formulas according to standard specifications	Risk of bias
Abdulmohsen et al[10] (2016)	0	1	2	2	1	Moderate
Rayyan et al[24] (2015)	1	0	2	2	1	Moderate
Penate et al[25] (2015)	2	0	0	2	1	Moderate
Thompson and Luo[13] (2014)	0	1	2	2	0	Moderate
Yanikoğlu et al[23] (2014)	0	1	2	2	1	Moderate
Hamza et al [30] (2014)	0	1	1	2	0	Moderate
Poonacha et al[14] (2013)	0	1	2	2	0	Moderate
Jo et al[9] (2011)	0	1	2	2	1	Moderate
Alt et al[20] (2011)	0	1	2	2	1	Moderate
Zortuk et al[15] (2010)	1	1	2	2	1	Moderate
Nejatidanesh et al[7] (2009)	1	1	2	2	0	Moderate
Balkenhol et al[21](2009)	0	1	2	2	0	Moderate
Balkenhol et al[18] (2008)	0	1	2	2	0	Moderate
Kim and Watts[11] (2007)	0	1	2	2	1	Moderate
Akova et al[22] (2006)	0	2	2	2	1	Moderate
Kim and Watts[27] (2004)	0	1	2	2	0	Moderate
Hamza et al[28] (2004)	0	1	2	2	0	Moderate
Yap et al[12] (2004)	0	1	2	2	1	Moderate
Lang et al[16] (2003)	1	1	2	2	1	Moderate
Haselton et al[8] (2002)	0	1	2	2	1	Moderate
Ireland et al[19] (1998)	1	1	2	2	1	Moderate
Samadzade et al[29] (1997)	1	1	2	2	1	Moderate
Gegauff and Wilkerson[26] (1995)	0	1	2	2	0	Moderate
Diaz-Arnold et al[17] (1990)	0	1	2	2	1	Moderate

The risks of bias most commonly found in the studies were: blinding of the testing machine operator, description of the sample size calculation, single operator of the machine and random assignment of specimens. The scores for blinding of the testing machine operator and description of the sample size calculation were generally low.

### Meta-analyses

Seven studies met the best requirement features for quantitative analysis. Eight meta-analyses, including three global and five subgroup analyses, were performed on the flexural strength, fracture toughness and hardness data.

#### Flexural strength

**Fig 3:**
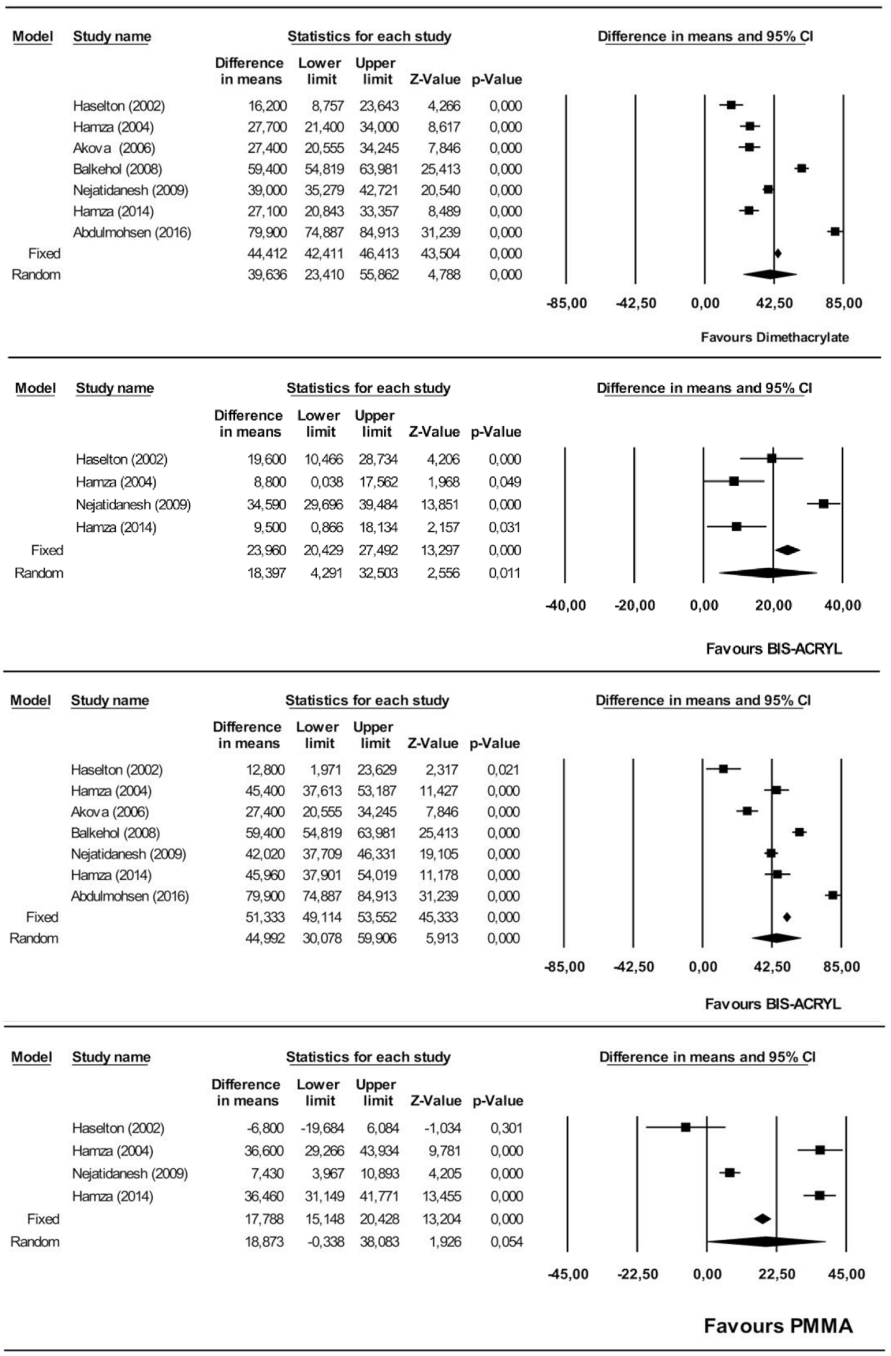
Forest plot of flexural strength. Flexural strength. Dimethacrylate vs. monomethacrylate groups (Fig 3A), Flexural strength. Bis-acryl vs. PMMA (Fig 3B), Flexural strength. Bis-acryl vs. PEMA (Fig 3C), Flexural strength. PMMA vs. PEMA (Fig 3D).

The dimethacrylates group showed significantly higher flexural strength than the monomethacrylates group, by an estimated 39.6 MPa (95% CI 23.4–55.8). The heterogeneity of the 7 studies included in the meta-analysis was high (Q = 377.1, heterogeneity p = 0.000, I^2^ = 98.4%). (Fig 3A).

The flexural strength of bis-acryl was estimated as significantly higher than that of PMMA, by 18.4 MPa (95% CI 4.3–32.5). The heterogeneity was high (Q = 41.2, heterogeneity p = 0.000, I^2^ = 92,7%) (Fig 3B).

On comparing bis-acryl with PEMA, the flexural strength of bis-acryl was significantly higher, by an estimated 44.9 MPa (95% CI 30.1–59.9). The heterogeneity was high (Q = 254.1, heterogeneity p = 0.000, I^2^ = 97.6%) (Fig 3C).

On comparing PMMA and PEMA, PMMA was not significantly different although the difference was estimated at 18.3 MPa (95% CI -0.33–38.1). The heterogeneity was high (Q = 121.1, heterogeneity p = 0.000, I^2^ = 97.5%) (Fig 3D).

#### Fracture toughness

**Fig 4:**
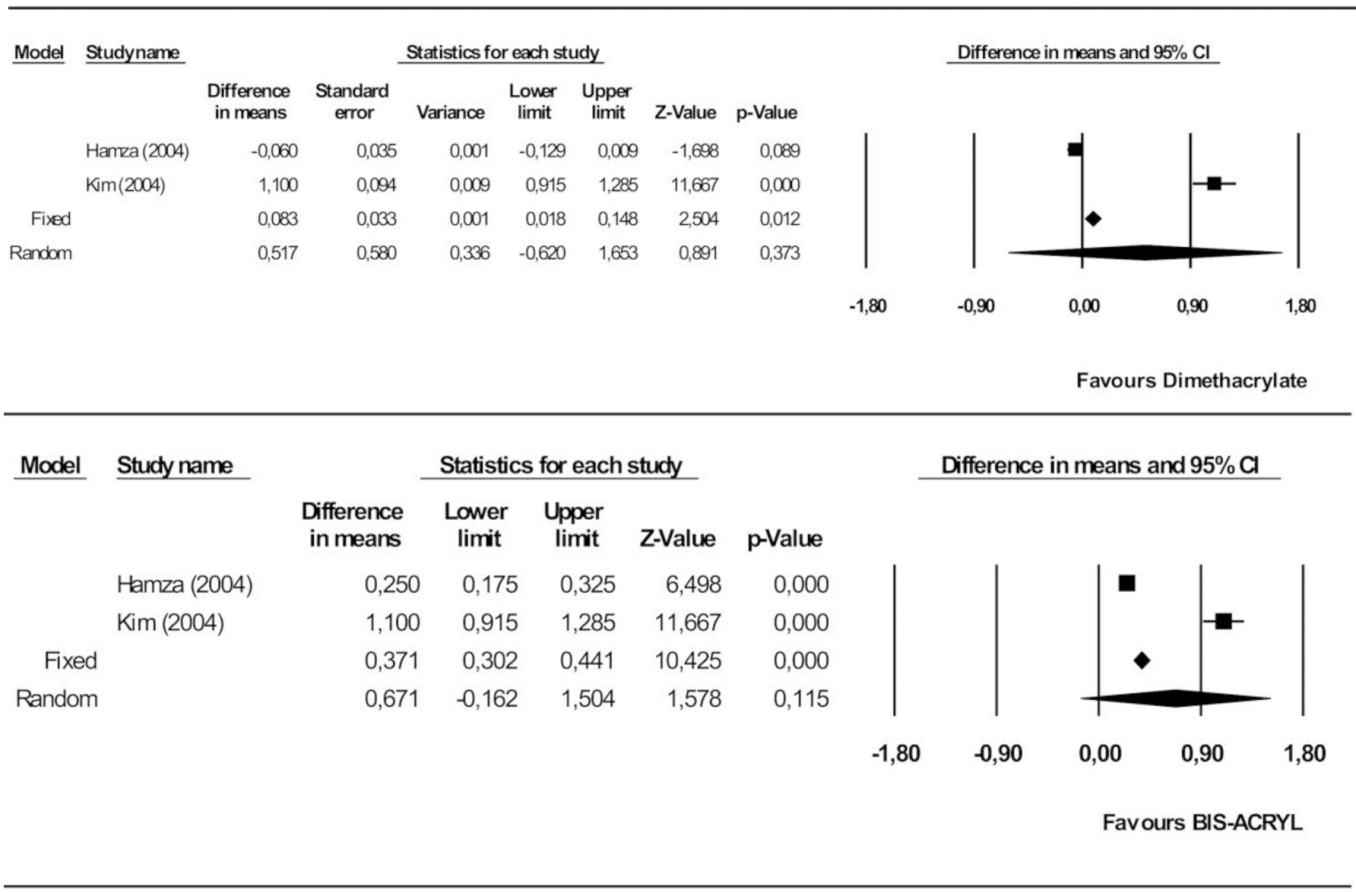
Forest plot of fracture toughness. Dimethacrylate vs. monomethacrylategroups (Fig 4A), Bis-acryl vs. PEMA (Fig 4B).

The random models analysis showed no significant difference between the dimethacrylate and monomethacrylate groups. The mean difference was estimated at 0.52 MPa.m^1/2^ in favor of dimethacrylate (95% CI 0.62–1.65). The heterogeneity of the studies included in the meta-analysis was high (Q = 132.7, heterogeneity p = 0.000, I^2^ = 99.2%) (Fig 4A).

No significant differences were found on comparing bis-acryl and PEMA. The effect was estimated at 0.67 MPa.m^1/2^ in favor of bis-acryl (95% CI -0.16–1.50). The heterogeneity was high (Q = 69.6, heterogeneity p = 0.000, I^2^ = 98,6%) (Fig 4B).

#### Knoop hardness

**Fig 5:**
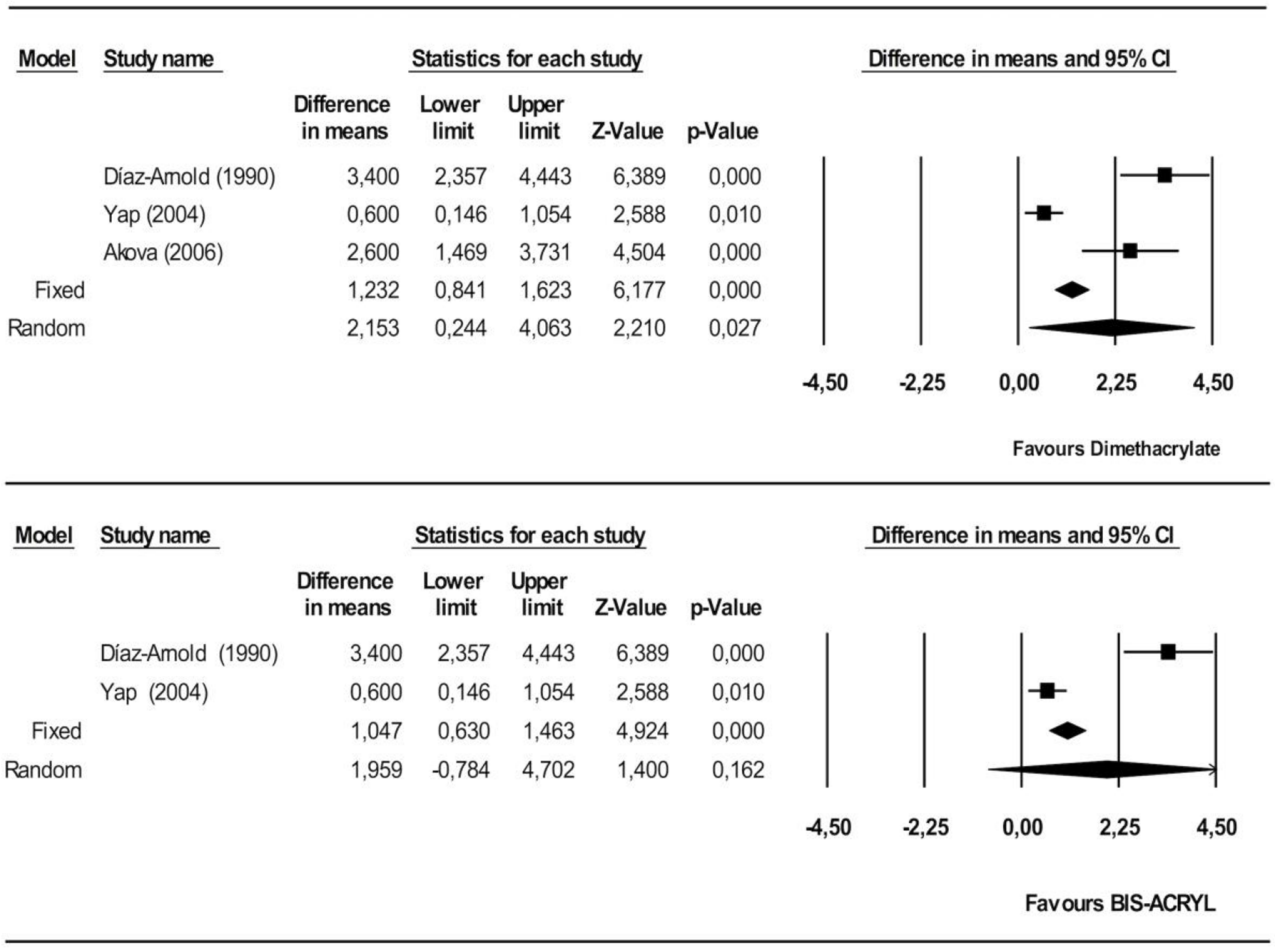
Forest plot of Knoop hardness. Dimethacrylate vs. monomethacrylate groups (Fig 5A), Bis-acryl vs. PMMA (Fig 5B).

Dimethacrylates proved significantly superior to monomethacrylates, by 2.15 KHN (95% CI 0.24–4.06). The heterogeneity of the studies included in the meta-analysis was high (Q = 29.6, heterogeneity p = 0.000, I^2^ = 93.3%) (Fig 5A).

Lastly, bis-acryl was not significantly superior to PMMA (1.44 KHN, 95% CI 0.95–1.93). The observed heterogeneity was high (Q = 23.2, heterogeneity p = 0.000, I^2^ = 95.7%) (Fig 5B).

As regards checking for publication bias impact, the following values were found through the classic fail-safe number: flexural strength comparison between the dimethacrylates group and the monomethacrylates group = 2941, between PMMA and bis-acryl = 125, between bis-acryl and PEMA = 3059, and between PMMA and PEMA = 178; fracture toughness comparison between the dimethacrylates group and the monomethacrylates group = 116, and between PEMA and bis-acryl = 185; Knoop hardness comparison between the dimethacrylates group and the monomethacrylates group = 45, and between PMMA and bis-acryl = 31.

## Discussion

A number of in vitro studies have been conducted to assess the mechanical properties of provisional restoration materials used with direct techniques in dentistry. No randomized controlled clinical trials (RCT) or systematic reviews appear to exist to assist in selecting the ideal material for specific clinical situations, leaving the choice of material to criteria such as ease of handling and time taken in the office [7]. The present systematic review and meta-analysis is the first to analyze and combine published data from in vitro studies in order to assess their combined effect and answer the question of whether, in provisional restorations fabricated by direct techniques, the dimethacrylate and monomethacrylate groups differ significantly in their mechanical properties in terms of flexural strength, fracture toughness and hardness.

The overall results of this meta-analysis show that dimethacrylate-based materials exhibit better mechanical responses in terms of flexural strength and hardness than monomethacrylate-based materials. In general, the basic chemical composition of dimethacrylates gives them better mechanical behavior against applied stresses, as they have a rigid, cross-linked structure owing to the presence of highly viscous and voluminous multifunctional monomers (Bis-GMA or TEGDMA) that can cross-link with other polymeric chains. This cross-linking, combined with inorganic loading, makes these materials strong and easy to handle and to polish, resulting in a low exothermic temperature [7–11]. In contrast, monomethacrylates are polymers composed of monofunctional molecules with a linear structure and low molecular weight, the lack cross-linking leading to lower rigidity and mechanical resistance [7,8,12].

The present results match those of some other studies [8,12–15] which suggest that as well as the basic chemical composition of the dimethacrylate group, factors such as individual formulation, supplements added by the manufacturer and the degree of polymerization and of cross-linking of the matrix influence the mechanical performance of the material. For instance, replacement of the rigid monomers of the first-generation materials by the elastic monomers currently being used has improved the ability to withstand high forces, as they exhibit a small elastic deformation before breaking [8,16]. In addition, the present study has found that auto-polymerizing materials in the dimethacrylates group exhibit greater flexural strength and hardness than those which are polymerized by light, owing to the greater presence of filler particles [9,17]. Compared to auto-polymerizing dimethacrylates, dual-cure dimethacrylates present flexural strength advantages for up to 72 hours, owing to the large amount of initial polymerization caused by the reaction to photo-initiation. After that time, the values are similar[7,18,19].

In the monomethacrylates group, the present meta-analysis found that PMMA presented greater flexural strength than PEMA. PMMA was not significantly different although the difference was estimated at 18.3 MPa in favour to PMMA. In this group, it is also important to understand that mechanical behavior is influenced by the individual formulation of each comercial brand. Provisional restoration based on PMMA have many advantages, in terms of strength, aesthetics and colour stability, marginal fit, and they can be easily fabricated, polished and repaired. However, it has been reported to have some serious disadvantages like irritation of vital tissues, which could be due to leaching of the free monomer, high polymerization exotherm during setting, low wear resistance and high volumetric shrinkage, and should be used for the indirect technique of TCB fabrication, or as PMMA shells that are fabricated in the laboratory, which are then lined in the patient’s mouth with PEM/monomer system [10].

PEMA based provisional restoration are suitable for both direct and indirect techniques, due to their minimal polymerization exotherm, low shrinkage and better biocompatibility compared with PMMA. However, due to PEMA resins being mechanically weaker and with less colour stability than PMMA, their use is limited to making posterior interim prostheses, in short-term provisional treatment. Most provisional restoration based on PEMA contain either the monomer isobutyl methacrylate (IBMA) or n-butyl methacrylate (nBMA). The latter was introduced as a new provisional restorative material due to having low water uptake, low polymerization exothermic reaction and low irritation to the vital tissue. [10] The former commercial PEMA based provisional material with IBMA monomer (e.g. Trim, Bosworth) contains a plasticizer (di-butyl phthalate, DBP), to improve handling properties and reduce setting time. The phthalate is not chemically bonded to the plasma network, and phthalates are considered endocrine disruptors chemicals that cause estrogenic behavior and are possible carcinogens [10]. Therefore ingesting a small amount of these elements may cause considerable problems to the living system [16]. Moreover, the plasticizer reduces the glass transition temperature (Tg) of polymers by weakening the links between the polymer chains and increasing their movements, since the plasticizer slowly leached out of the material, and in turn contributed to more water being absorbed by the matrix to fill the spaces left by the leached material. Therefore, these materials have a lower flexural strength.[10].

Flexural strength and fracture toughness of provisional restorations in the monomethacrylates group decrese gradually in time. At the initial stages, the effect of water in monomer hydrolysis is low or nonexistent [13,18,20], allowing the material a certain degree of plastic deformation before breaking, known as ductile behavior. When the storage time is increased, however, this group of materials tends to absorb water, owing to its linear polymer network structure, the high polarity of its molecules and the air bubbles immersed in its structure as a result of manual mixing [7,8]. This water absorption leads to hydrolysis of the monomers, giving rise to a constant decrease in its mechanical properties [9,10,18,21].

In contrast, dimethacrylates have a rigid central structure that allows them to absorb only 0.8% by weight of water, it is due to the increase in the conversion of reactive double bonds by radicals and because the relaxation phenomena take place within the polymer network. In addition, the self-mixing cartridge system makes it possible to control the proportions of the components and avoid air entrapment [8]. However, thermocycling weakens the internal structure of the material, because the increasing water absorption, although this is less than in monomethacrylates, generates a reduction in the intermolecular forces between the chains of polymer. In addition, phenomena of hydrolysis of the silane layer surrounding the filler particles are observed, which promotes the propagation of cracks in the periphery of the filler particles.[22] These materials can withstand high forces before breaking, once the stress is greater than the proportional limit they fracture immediately rather than undergoing deformation. Consequently, they are described as rigid materials but brittle [9,10]. Other in vitro studies have assessed the mechanical effect that solutions which simulate foods have on the provisional restoration [12,22,23]. Heptane and 75% ethanol caused softening of the polymer matrix in both groups and degradation of the filler-matrix interface in the dimethacrylates group, but the dimethacrylates withstood breakdown by the solutions better than the monomethacrylates, owing to their cross-linked bi-functional monomers [12,22]. Only one study found that the solutions tested did not have a statistically significant effect on the flexural strength of the provisional restoration materials [23].

In the conventional direct technique, provisional restorations are in direct contact with saliva and intraoral moisture immediately after fabrication. This means that regardless of the basic chemical composition, the provisional restorations manually fabricated could suffer polymerization inhibition effects due to the oxygen, as the process of radical polymerization is still in progress, which affect long-term mechanical properties and color stability [21]. Nowadays, provisional restoration materials are available in blocks for machining by CAD/CAM systems. These blocks are fabricated under optimum polymerization conditions with no interference from water, giving adequate time for post-polymerization processes and relaxation phenomena, which means that the provisional restorations fabricated from blocks, whether monomethacrylate or dimethacrylate, have superior mechanical properties to those fabricated by conventional direct techniques from the moment the restoration is put in place [20,24,25].

The present meta-analysis also found that there was no significant difference between the dimethacrylates and monomethacrylates as regards their fracture toughness, in other words, their ability to stop cracks propagation. However, the way in which the cracks propagation are interrupt varies between the groups. In the dimethacrylate group, the presence of inorganic filler, the conversion of reactive double bonds during polymerization and relaxation phenomena within the polymer network, this renders the polymer network less sensitive to crack propagation. Although this ability is an advantage compared to the monomethacrylate group in the initial stages, it slowly diminishes owing to breakdown by water, which weakens the intermolecular forces in the polymer chain and hydrolyses the silane layer surrounding the filler particles, encouraging crack propagation at the edge of the filler particles as time passes [13,16,21,26,27]. In the monomethacrylate group, however, occurs a ductile fracture which differs from a brittle fracture in dimethacrylate group. Prior to fracturing, the specimen undergoes considerable plastic deformation, in this group the cracks are diverted by the plasticizing effect of water, which makes the fracture toughness acceptable only in the early stages [26], although it gradually diminishes over time because of water absorption by polymers which are not cross-linked, which weakens the material [21].

The structure of provisional restorations can be reinforced with fiberglass or polyethylene to improve their flexural strength and fracture toughness [27–29]. As regards flexural strength, reinforcement changes the fracture path: instead of catastrophic fractures (abutments and pontics), the fractures become partial and easy to repair (chipping on free surfaces), owing to the transfer of stresses from the weak polymer matrix to the fibers, which have high tensile resistance. As regards fracture toughness, the fibers bridge the crack and oppose its opening, exercising a closing force [29]. The reinforced specimens are as resistant as provisional restorations machined with CAD/CAM [25], and the stronger the reinforcing fiber’s adhesion to the polymer matrix, the greater the reinforcement effect, so silane-impregnated fiberglass provides greater reinforcement then plasma-impregnated polyethylene fibers [28,29]. Reinforcement by the addition of 1% by weight of polyhedral oligomeric silsesquioxane (POSS) to the formulation of the materials was also assessed. The results were not uniform and showed that the particular chemical composition of the provisional materials determines the ability of POSS to improve their mechanical properties [30].

The standards and specifications for in vitro testing of flexural strength employed in the studies included were ISO 4049:2000 [18], ISO 10477:2004 [13,26,27] and ANSI/ADA specification N° 27 [7,8,14,30]. Although their technical specifications are not very different from each other, ISO 10477:2004 considers criteria that are inherent to the process of provisionalization and the fabrication of provisional crowns and bridges; the optional addition to this standard is ANSI/ADA Standard No. 53 (ADA53-2013). There is no standard dental protocol for determining fracture toughness in polymeric materials [21]. The studies included in this review used ISO 13586 [21], ISO D256-97 [13], ASTM no. E 399–83 [26,28], and British Standard 5447[27]. The ISO 13586:2000 standard describes the assessment of fracture toughness in plastics assuming linear elastic fracture mechanics (LEFM), in which the plastic deformation zone remains concentrated around a small area at the point of the crack [21]. Two types of test were used to analyze hardness: Knoop and Vickers. The literature disagrees about which is the ideal test. Some authors [9,12,17,22] suggest that Knoop hardness tests allow elastic recovery of the material along the short axis, so determine the hardness values irrespective of the ductility of the test material. However, another study [13] suggests that Vickers hardness is a universal test, less sensitive to surface conditions, but more sensitive to measurement error.

## Limitations

Currently there is no validated, clearly established criterion for assessing the methodological quality and risk of bias of in vitro studies. However, the modified ARRIVE and CONSORT criteria [5] and an adaptation of the Cochrane risk-of-bias tool domains for systematic reviews taken from a previous study were used in the present review [6].

The studies included in the systematic review presented moderate to high methodological quality and moderate risk of bias, but the meta-analysis showed high data heterogeneity (>90%). This confirms their differences in methodology, with a high number of variables such as lack of experimental test standardization and absence of sample size calculation, machine operator blinding and calibration of the universal testing machine, generating a wide range of flexural strength, fracture toughness and hardness values with high standard deviations between the studies included. For this reason, we recommend the standardization of the methodology of in vitro studies following the guidelines of Academy of Dental Materials guidance for mechanical properties[31].

The present review summarizes in vitro data. Although some studies show that the external validity of in vitro tests to predict the clinical performance of dental materials is limited [32,33], a systematic review, found that there is a moderately positive correlation between clinical and laboratory outcomes, besides, fracture toughness being mostly correlated with clinical fracture and flexural strength with clinical wear[34]. Well designed in vitro studies need to be taken into account, owing to their ability to clarify any initial queries before conducting clinical trials [6].

The successful performance of a provisional material is not based exclusively on its mechanical properties but also on its interaction with its immediate environment, so other factors such as marginal adaptation, color stability, and pulp and gum response need to be assessed. For this reason, clinical studies should be conducted to lend greater external validity to the present findings.

## Conclusions

The available evidence after conducting this systematic review and meta-analysis indicates that dimethacrylate-based provisional restorations possess better mechanical behavior than monomethacrylate-based ones in terms of flexural strength and hardness, but there are no significant differences in fracture toughness. Among the monomethacrylates, PMMA shows greater flexural strength than PEMA.

In order to improve the quality of future studies, it would be advisable to conduct in vitro experimental testing with large samples, standardized specimen sizes, and blinded, calibrated testing machines in order to reduce the risk of bias.

## Supporting information

S1 Table PRISMA 2009 checklist. (PDF)

S2 Table Methodological characteristics of in-vitro studies based on arrive and consort modificated criteria. (DOCX)
