# Correction: Mechanical properties of provisional dental materials: A systematic review and meta-analysis

**DOI:** 10.1371/journal.pone.0196264

**Published:** 2018-04-17

**Authors:** Daniela Astudillo-Rubio, Andrés Delgado-Gaete, Carlos Bellot-Arcís, José María Montiel-Company, Agustín Pascual-Moscardó, José Manuel Almerich-Silla

Daniela Astudillo-Rubio should not be designated as corresponding author. The correct corresponding author is José María Montiel-Company. The listed email address (jose.maria.montiel@uv.es) is correct.
