# Supplementary material for: Mechanical properties of provisional dental materials: A systematic review and meta-analysis
Source: PLoS One. 2018 Feb 28;13(2):e0193162. doi: 10.1371/journal.pone.0193162 (PMC5830998; doi:10.1371/journal.pone.0193162)
Supplement: S2 Table — (DOCX) [file pone.0193162.s002.docx]

| **S2 Table.** Methodological characteristics of In-Vitro Studies based on Arrive and Consort modificated Criteria | | | | | | | | | | | | |
| --- | --- | --- | --- | --- | --- | --- | --- | --- | --- | --- | --- | --- |
|  | **Item (grade)** | | | | | | | | | | | |
| **Author, year** | **1**  **(0,1)** | **2**  **(1,2,3)** | **3**  **(1,2,3)** | **4**  **(1,2)** | **5**  **(1,2,3)** | **6**  **(1,2,3)** | **7**  **(1,2,3)** | **8**  **(1,2,3)** | **9**  **(1,2,3)** | **10**  **(0,1,2)** | **11 (0,1)** | **12 (0,1)** |
| Abdulmohsen et al[10]  (2016) | 1 | 3 | 3 | 2 | 2 | 2 | 1 | 3 | 3 | 1 | 0 | 1 |
| Rayyan et al[24]  (2015) | 1 | 2 | 3 | 2 | 2 | 2 | 2 | 2 | 2 | 1 | 0 | 1 |
| Penate et al[25]  (2015) | 1 | 3 | 3 | 2 | 2 | 3 | 3 | 3 | 3 | 1 | 0 | 1 |
| Thompson and Luo[13]  (2014) | 1 | 3 | 3 | 2 | 3 | 2 | 1 | 2 | 3 | 1 | 0 | 1 |
| Yanikoğlu et al[23] (2014) | 1 | 3 | 3 | 2 | 2 | 2 | 1 | 3 | 3 | 1 | 0 | 1 |
| Hamza et al [30]  (2014) | 1 | 2 | 3 | 2 | 2 | 2 | 1 | 2 | 2 | 1 | 0 | 1 |
| Poonacha et al[14]  (2013) | 1 | 2 | 2 | 2 | 2 | 2 | 1 | 2 | 2 | 1 | 1 | 1 |
| Jo et al[9]  (2011) | 1 | 3 | 3 | 1 | 1 | 3 | 1 | 2 | 2 | 2 | 0 | 1 |
| Alt et al[20]  (2011) | 1 | 2 | 3 | 1 | 3 | 2 | 1 | 3 | 3 | 2 | 0 | 1 |
| Zortuk et al[15]  (2010) | 1 | 2 | 2 | 1 | 2 | 3 | 1 | 3 | 3 | 1 | 1 | 1 |
| Nejatidanesh et al[7]  (2009) | 1 | 3 | 3 | 2 | 2 | 3 | 2 | 2 | 3 | 2 | 0 | 1 |
| Balkenhol et al[21]  (2009) | 1 | 2 | 3 | 2 | 3 | 3 | 1 | 3 | 3 | 2 | 0 | 1 |
| Balkenhol et al[18]  (2008) | 1 | 2 | 3 | 2 | 3 | 3 | 1 | 3 | 3 | 2 | 0 | 1 |
| Kim and Watts[11]  (2007) | 1 | 3 | 3 | 2 | 3 | 2 | 1 | 2 | 1 | 1 | 0 | 1 |
| Akova et al[22] (2006) | 1 | 3 | 3 | 2 | 2 | 3 | 1 | 2 | 3 | 2 | 0 | 1 |
| Kim and Watts[27]  (2004) | 1 | 3 | 3 | 2 | 3 | 3 | 1 | 2 | 2 | 1 | 0 | 1 |
| Hamza et al[28] (2004) | 1 | 3 | 3 | 2 | 3 | 3 | 1 | 3 | 3 | 2 | 0 | 1 |
| Yap et al[12]  (2004) | 1 | 3 | 2 | 2 | 2 | 3 | 2 | 3 | 3 | 1 | 0 | 1 |
| Lang et al[16]  (2003) | 1 | 2 | 2 | 2 | 1 | 2 | 1 | 2 | 2 | 1 | 0 | 0 |
| Haselton et al[8] (2002) | 1 | 3 | 3 | 2 | 2 | 3 | 1 | 3 | 3 | 2 | 0 | 1 |
| Ireland et al[19]  (1998) | 1 | 2 | 2 | 2 | 2 | 2 | 1 | 2 | 3 | 1 | 0 | 1 |
| Samadzade et al[29]  (1997) | 1 | 2 | 2 | 2 | 1 | 2 | 1 | 2 | 2 | 1 | 0 | 1 |
| Gegauff and Wilkerson[26] (1995) | 1 | 2 | 3 | 2 | 3 | 2 | 1 | 2 | 1 | 1 | 1 | 1 |
| Diaz-Arnold et al[17]  (1990) | 1 | 2 | 2 | 1 | 2 | 2 | 1 | 1 | 2 | 0 | 0 | 1 |
